# CAPRIN1 Is Required for Control of Viral Replication Complexes by Interferon Gamma

**DOI:** 10.1128/mbio.00172-23

**Published:** 2023-04-13

**Authors:** Chaitanya Kurhade, Soowon Kang, Scott B. Biering, Seungmin Hwang, Glenn Randall

**Affiliations:** a Department of Microbiology, The University of Chicago, Chicago, Illinois, USA; b Division of Infectious Diseases and Vaccinology, School of Public Health, University of California, Berkeley, Berkeley, California, USA; c Department of Pathology, The University of Chicago, Chicago, Illinois, USA; National Institutes of Health

**Keywords:** autophagy, interferon gamma, replication compartments

## Abstract

Replication complexes (RCs), formed by positive-strand (+) RNA viruses through rearrangements of host endomembranes, protect their replicating RNA from host innate immune defenses. We have shown that two evolutionarily conserved defense systems, autophagy and interferon (IFN), target viral RCs and inhibit viral replication collaboratively. However, the mechanism by which autophagy proteins target viral RCs and the role of IFN-inducible GTPases in the disruption of RCs remains poorly understood. Here, using murine norovirus (MNV) as a model (+) RNA virus, we show that the guanylate binding protein 1 (GBP1) is the human GTPase responsible for inhibiting RCs. Furthermore, we found that ATG16L1 mediates the LC3 targeting of MNV RC by binding to WIPI2B and CAPRIN1, and that IFN gamma-mediated control of MNV replication was dependent on CAPRIN1. Collectively, this study identifies a novel mechanism for the autophagy machinery-mediated recognition and inhibition of viral RCs, a hallmark of (+) RNA virus replication.

## INTRODUCTION

Positive-strand RNA viruses are important human pathogens that infect millions of people worldwide (1). A common feature of all (+) RNA viruses is the modification of cytosolic endomembranes, such as the endoplasmic reticulum (ER), to form RCs (2). The RC forms a platform for RNA replication and protects the replicating RNA from detection by innate immune host pattern recognition receptors (3). While the mechanisms by which viruses form RCs are well studied (4), considerably less is known about the host response to RCs.

Autophagy is the cellular recycling machinery that sequesters intracellular constituents into double-membrane-bound autophagosomes, which deliver cargo to lysosomes for degradation (5). Efficient autophagosome formation and selective capture of cargo involve the covalent attachment of microtubule associated protein 1 light chain 3 (LC3) to phosphatidylethanolamine (PE) on the surface of the phagophore membrane (6). The lipidation of LC3 on the phagophore membrane involves multiple steps analogous to ubiquitin conjugation to a target protein. An E1-like activating enzyme, ATG7, and an E2-like conjugating enzyme, ATG3, function together with an E3-like ligase, the ATG12–ATG5-ATG16L1 complex, to conjugate LC3 to PE (7). Intriguingly, the LC3 conjugation machinery is required for the inhibition of the (+) RNA virus MNV by interferon gamma (IFNG), which specifically targets the replication stage of viral life cycle (8, 9). LC3 accumulates at viral RCs in MNV-infected cells in an ATG5 and ATG16L1-dependent manner (8, 9); then, IFN-inducible GTPases, including immunity-related GTPases (IRGs) and GBPs, target the LC3-marked RCs upon their induction by IFNG, resulting in the inhibition of viral replication (8, 9). We found the same role of autophagy machinery in controlling parasitophorous vacuole membrane of Toxoplasma gondii (10) and termed this process **T**argeting by **A**utopha**G**y proteins (TAG) (11). However, it remains unknown how the ATG12–ATG5-ATG16L1 complex detects RC and recruit GTPases to the replication site.

IFNG is a multifunctional cytokine that regulates a broad spectrum of immune networks (12). In contrast to the type I and III interferons produced by any cells upon viral infection, IFNG expression is restricted to specific subsets of immune cells upon infection or stimulation with antigens, but almost all cells can respond to IFNG (13). Although IFNG has now been shown to play diverse roles in immune modulation and defense against a broad-spectrum of pathogen, it was originally discovered, and named, as a soluble factor that interferes with viral replication (14). In addition to immune modulating function, which mediates broad immune defense against infection, IFNG can play a role in direct antiviral effects on infected cells and neighboring cells (15). By inducing multiple downstream interferon-inducible genes and targeting specific viral components, IFNG has shown direct antiviral activity and can target different stage of virus life cycle to inhibit virus infection. Despite the observations about IFNG direct antiviral activity, the detailed mechanisms of IFNG have not been well characterized.

In this study, we investigated two aspects of the TAG system: (i) Do the human homologs of the mouse GBPs also have antiviral activity via TAG and what functional domains are required for TAG? (ii) How does the LC3 conjugation machinery recognize MNV RCs? We observed a conserved role for mGBP2/hGBP1 in TAG antiviral activity. We also discovered a role for WIPI2 recruitment of ATG16L1 to RCs and uncovered a novel role for CAPRIN1 in recruiting ATG16L1 to RCs.

## RESULTS

### Human GBP1 localizes to the MNV replication complex and is sufficient for IFNG-mediated control of MNV.

We previously showed that human GBPs target MNV RCs upon their formation in human cells (8). There are 23 IRGs, 11 GBPs, and two pseudogenes in the mouse, and mouse IRGs play a crucial role in GBP function. In contrast, only 2 IRGs and 7 GBPs have been identified in humans, in which hIRGs are not induced by IFNG (16). To understand the antiviral mechanism of human GBPs, we investigated which hGBP plays a crucial role against MNV.

**FIG 1:**
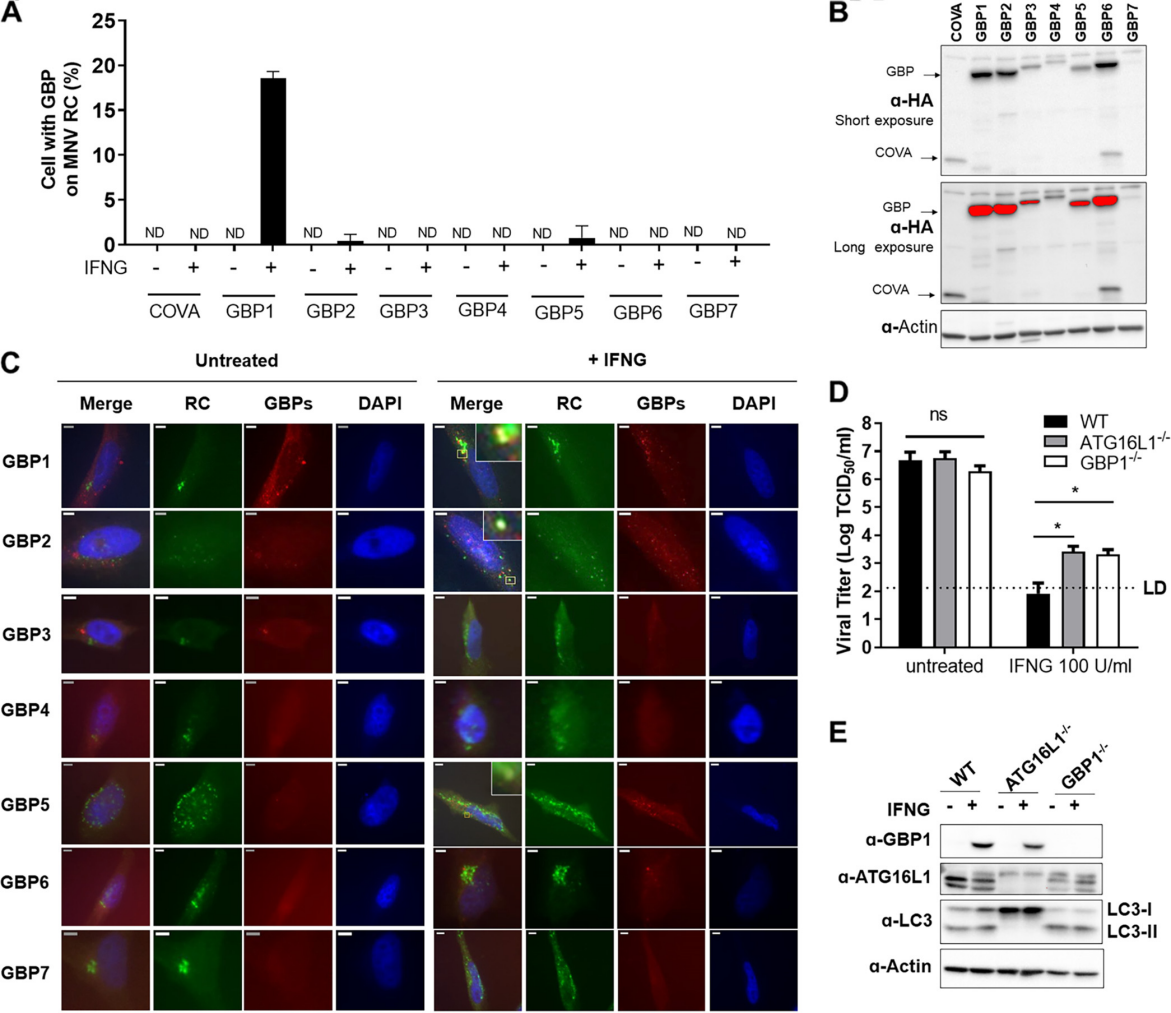
Human GBP1 localizes with the MNV replication complex and is important for IFNG-mediated control of MNV replication. (A) Immunofluorescence assay (IFA) for the localization of GBPs to the MNV RC in HeLa cells expressing individual GBPs. The cells were transfected with a plasmid expressing MNV ORF1 for 6 h followed by treatment without or with IFNG at 100 U/mL for an additional 18 h. Quantitation of IFA for the localization of GBPs (1–7) on to the MNV RC is shown (B) Representative Western blot of HeLa cells from (A); expressing COVA (cytoplasmic ovalbumin; used as a control) and individual GBPs. (C) IFA for the localization of GBPs to the MNV RC. HeLa cells expressing individual GBPs were transfected with a plasmid expressing MNV ORF1 for 6 h followed by treatment without or with IFNG at 100 U/mL for an additional 18 h. Representative images shown here. Zoomed images shown only for cells showing colocalization. Scale bar, 5 μm. (D) As human cells do not express MNV receptor, MNV vRNA were transfected into the WT, ATG16L1^−/−^ and GBP1^−/−^ HeLa cells and MNV production was measured by TCID50. (E) Representative Western blot of HeLa cells for (D); WT, ATG16L1^−/−^ and GBP1^−/−^. Cells were untreated or treated with 100 U/mL IFNG for 24 h. Data were analyzed using *unpaired t test*. ND, not detected; ns, not significant; *, *P* < 0.05. Dashed lines in D indicate the limit of detection (LD).

We examined the localization of all seven human GBPs individually, with or without IFNG stimulation. We used HeLa cell line expressing individual HA-tagged GBPs or COVA (cytoplasmic ovalbumin) which was used as a negative control. We transfected these cells with a plasmid expressing MNV ORF1 for 6 h followed by treatment with or without IFNG at 100 U/mL for an additional 18 h and tested colocalization of HA-GBPs with the MNV RC. Only HA-GBP1 showed significant colocalization with MNV RC in the presence of IFNG, suggesting that an IFNG-induced cofactor is necessary for targeting GBP1 to the viral RC (Fig. 1A to C). There was various expression of HA-GBP proteins in transduced HeLa cells, with GBP7 having the lowest expression compared to other GBPs (Fig. 1B). Although the differential GBP expression should be considered when interpreting the experiment, HA-GBP1 clearly colocalizes with MNV RC to significantly higher levels than other GBPs. We also observed a significant reduction of IFNG control of MNV replication in *GBP1*^−/−^ and *ATG16L1*^−/−^ HeLa cells compared to WT cells (Fig. 1D and E), demonstrating that ATG16L1 (as already shown in [8]) and GBP1 function in IFNG control of MNV replication in human cells.

**FIG 2:**
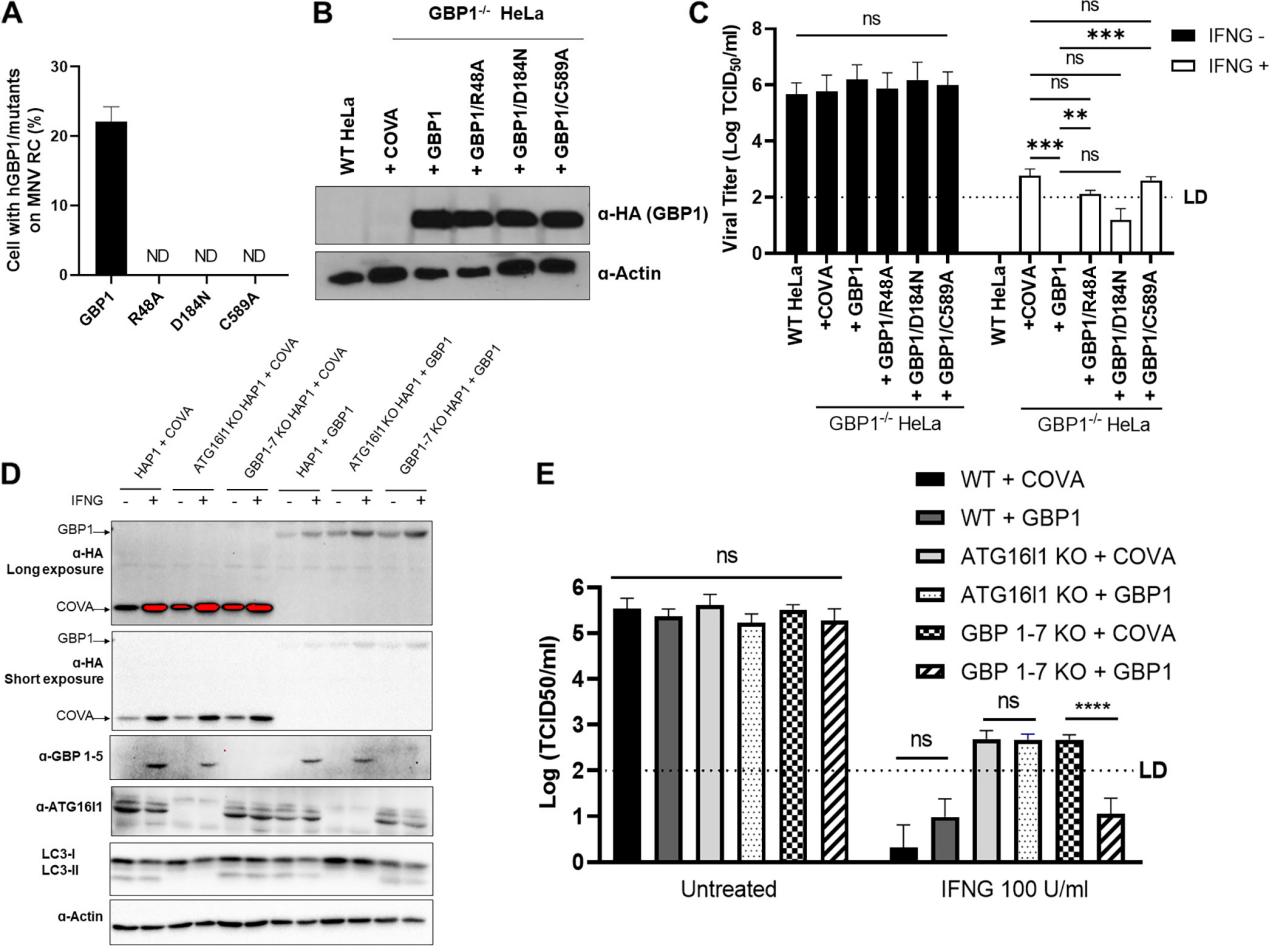
GBP1 is the necessary and sufficient human GBP for IFNG inhibition of MNV replication. (A) Quantitation of IFA for the localization of GBP1 mutants on to the MNV RC. HeLa cells expressing GBP1 and mutants were transfected with a plasmid expressing MNV ORF1 for 6 h followed by treatment with IFNG at 100 U/mL for an additional 18 h. After incubation, the cells were fixed and stained with anti-Propol antibody and anti HA antibody for GBP1 and its mutants (B) Representative Western blot of HeLa cells to confirm the protein reconstitution for (A and C). (C) MNV production in the WT and GBP1^−/−^ HeLa cells expressing either COVA (control) or WT/mutants GBP1. (D) Representative Western blot of WT, ATG16L1^−/−^ and GBP1-7^−/−^ HAP1 cells expressing either COVA (control) or GBP1 for (E). Cells were untreated or treated with 100 U/mL IFNG for 24 h (E) MNV production in the WT, ATG16L1^−/−^ and GBP1-7^−/−^ HAP1 cells expressing either COVA (control) or GBP1. For all MNV production measurement in HeLa cells, each group of cells were untreated or treated with the 100 U/mL of IFNG for 24 h and then transfected with 50 ng MNV viral RNA. At 24 hpt, cells were harvested to titer infectious virus by TCID_50_. Data were analyzed using *unpaired t test*. ND, not detected; ns, not significant; **, *P* < 0.01; ***, *P* < 0.001; ****, *P* < 0.0001. Dashed lines in C, D indicate the limit of detection (LD). ND, Not Detected.

GBPs belong to the dynamin family, with nucleotide-dependent oligomerization and GTPase activity, and has a C-terminal isoprenylation anchor that enables membrane binding. To determine which GBP1 functions are responsible for targeting and inhibiting MNV RC, we generated various GBP1 mutants, including GTP hydrolysis-defective (GBP1/R48A), GTP binding-deficient (GBP1/D184N), and membrane association impaired (GBP1/C589A) (17, 18). While all GBP1 mutants were expressed at comparable levels, they failed to target the MNV RC formed by transfecting MNV ORF1 into HeLa cells (Fig. 2A and B). Knockout (KO) of GBP1 interfered with the antiviral function of IFNG, and IFNG control of MNV replication was restored when GBP1 expression was reconstituted. However, reconstitution of GBP1 expression with either the GBP1 mutants or the negative control failed to completely restore IFNG antiviral activity (Fig. 2C). These results suggest that the known intrinsic properties of GBP1 are required for targeting to viral RC and inhibition of MNV replication by IFNG.

The GBPs have similarities with dynamin-like GTPases and undergo self-assembly to form large complexes. In the case of several GBPs, including GBP1, prenylation of a C-terminal CaaX motif enables an association to the membrane. The GBPs can also heterodimerize with other members of the GBP family resulting in hierarchical positioning on the intracellular vesicles and recruiting nonprenylated GBPs to their subcellular compartments (17, 19, 20). These properties raise the question of whether GBP1 alone plays a role in GBP1 function in IFNG-mediated control of MNV or whether other GBPs also participate. We previously observed that MNV replication was substantially less inhibited by IFNG in human HAP1 cells with *ATG16L1*^−/−^ or *GBP1-7*^−/−^ (whole GBP KO [21]) than in the control cells (8). GBP1 expression in *ATG16L1*^−/−^ HAP1 cells cannot rescue IFNG-mediated control of MNV, as expected, because it lacks the LC3 conjugation system required for GBP targeting to the MNV RC (Fig. 2D and E). In contrast, GBP1 expression in *GBP1-7*^−/−^ HAP1 cells restored the IFNG-mediated inhibition of MNV replication. Taken together, these results show that GBP1 is the necessary and sufficient human GBP for IFNG inhibition of MNV replication.

### ATG16L1 colocalizes with MNV and HNV NS4-derived RCs.

**FIG 3:**
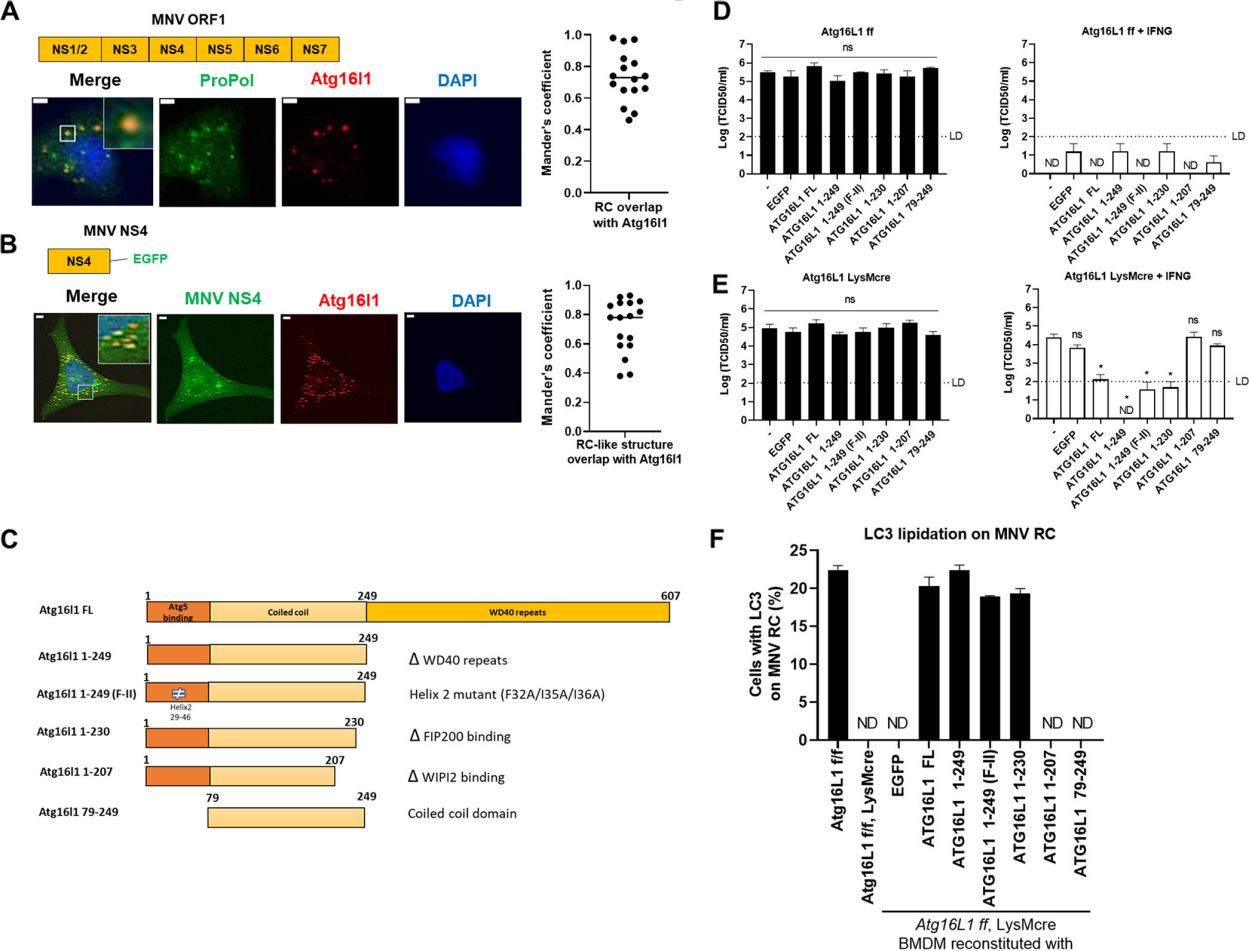
ATG16L1 localizes to the RCs formed by MNV protein expression and WIPI2 binding region of ATG16L1 (208-230) is required for IFNG-mediated inhibition of MNV replication. (A) IFA for the localization of ATG16L1 to viral RC (via anti-Propol) in HeLa cells. The cells were transfected with MNV ORF1 and pDRFP-ATG16L1 plasmid and incubated for 24 h. After incubation, the cells were fixed and stained with anti-Propol antibody. (B) IFA for the localization of ATG16L1 to viral RC-like compartment formed by NS4 in HeLa Cells. The cells were transfected with MNV NS4-EGFP and pDRFP-ATG16L1 plasmid and incubated for 24 h. After incubation, the cells were fixed. Representative images (A and B, left) and Mander’s coefficients from 2 experiments (A and B, right) are shown. (C) Schematic overview of wild type and mutants ATG16L1 indicating functional regions and mutation. (D and E) MNV production in *Atg16l1^f/f^* control BMDM (D) and *Atg16l1^f/f^+LysMcre* BMDM (E). BMDM cells were untransduced (-, control) or transduced with control (EGFP) or WT/mutants ATG16L1s described in (C). After completing BMDM differentiation (25 days), cells were untreated or treated with 100 U/mL IFNG for 24 h and then infected with MNV at MOI of 0.05. The cells were harvested at 24 hpi to titer infectious virus by TCID_50_ assay. (F) IFA for the localization of LC3 on to the MNV RC (Propol) at 10 hpi in *Atg16l1^f/f^* and *Atg16l1^f/f^+LysMcre* BMDM. The cells were untransduced or transduced with control (EGFP) or WT/mutants form of ATG16L1. Quantitation for the localization of LC3 to the MNV RC are shown. Data were analyzed using One-way ANOVA with Tukey’s multiple-comparison test. ND, not detected; ns, not significant; *, *P* < 0.05. In Figure D and E dashed line indicates the limit of detection (LD).

Previously, we found that individual expression of ATG16L1, but not ATG5 or ATG12, is sufficient to localize to the MNV RC, suggesting that it interacts with a component of the RC and recruits the ATG12–ATG5-ATG16L1 complex to the RC (9). ATG16L1 is a well-known scaffold protein that primarily functions through protein-protein interactions (22). Therefore, we hypothesized that the interaction of ATG16L1 with other protein(s) would be required for the viral RC targeting of ATG16L1. We observed that ATG16L1 is localized to the RC formed upon MNV ORF1 expression (9) (Fig. 3A), consistent with our previous report that LC3 localizes with RC-like structures formed by MNV ORF1 expression (8). We also observed that expression of either MNV or human Norwalk virus (HNV) NS4, a component of ORF1 that is sufficient to induce RC-like structures (23), can colocalize with ATG16L1 (Fig. 3B and Fig. S1A). In addition, LC3 and GBP1-5 also colocalized with MNV NS4 puncta (Fig. S1B).

10.1128/mbio.00172-23.1 FIG S1 ATG16L1 localization to RCs. (A) IFA for localization of GBPs and LC3 to viral RC-like compartment formed by MNV NS4. Hela cells were transfected and incubated with MNV NS4-EGFP plasmid for 24 hrs. Following 24 hrs incubation, the cells were stained with antibodies for GBP1-5 and LC3. Representative images (A, top) and Mander’s coefficients and percentage of cells with GBP1-5 on RC or and percentage of cells with LC3 on RC (± SEM) from 2 experiments (A, bottom) are shown. (B) IFA for the localization of ATG16L1 to viral RC-like compartment formed by HNV NS4. Hela cells transfected and incubated with HNV NS4-EGFP and pDRFP Atg16L1 plasmid for 24 hrs. Following 24 hrs, the cells were fixed and analyzed. Representative images (B, left) and Mander’s coefficients (± SEM) from 2 experiments (B, right) are shown. (C) A representative western blot data of cells described in Fig. 3D and 3E, indicating transduced protein expression (HA), ATG16L1 depletion status of BMDM (ATG16L1, LC3), and actin as loading control (*n* = 2). The red arrowhead indicates protein expression of indicated construct with respect to its protein size. (D) Representative images of IFA for the localization of LC3 on to the MNV RC and its quantification as shown in Fig. 3F. Download FIG S1, TIF file, 0.7 MB. Copyright © 2023 Kurhade et al. 2023 Kurhade et al. https://creativecommons.org/licenses/by/4.0/ This content is distributed under the terms of the Creative Commons Attribution 4.0 International license.

### The ATG16L1 WIPI2-binding domain is required for IFNG control of MNV infection.

ATG16L1 is recruited to distinct subcellular location to promote LC3 lipidation by interacting with different protein partners (24–27). ATG16L1 consists of three domains: ATG5 binding region, coiled-coil domain, and WD-40 repeats (Fig. 3C). It can bind directly to membranes through N- and C-terminal membrane-binding regions, which is essential for LC3B lipidation and for VPS34-independent LC3 lipidation on endosomes, respectively (24). Recent studies have shown that during starvation, membrane recruitment of ATG16L1 involves its binding to WIPI2B, which binds phosphatidylinositol-3-phosphate (PI3P) (25). WIPI2B has also been shown to bind the phagophore membrane surrounding Salmonella, which recruits ATG16L1, initiates LC3 lipidation, autophagosome formation, and engulfment of Salmonella to restrict its proliferation (25). FIP200, another interacting partner with ATG16L1, recruits it to the isolation membrane (27, 28).

We generated truncation mutants of ATG16L1 to identify important domains for ATG16L1 targeting to viral RCs; full length (FL), ΔWD40 repeats (1-249), F32A/I35A/I36A that is defective for the direct membrane binding capacity (1-249-FII), ΔFIP200 binding (1-230), ΔWIPI2 binding (1-207), and the coiled-coil domain (79-249) (Fig. 3C). We transduced these constructs in *Atg16l1^f/f^* (*Atg16l1* WT) and *Atg16l1^f/f^* + *LysMcre* (*Atg16l1* KO in myeloid cell lineage) bone marrow-derived macrophage (BMDM) cells and measured the restoration of IFNG-mediated control of MNV. In *Atg16l1* WT BMDMs, ATG16L1 expression did not affect the MNV control by IFNG, similar to the untransduced and EGFP-transduced negative controls (Fig. 3D and S1C). In *Atg16l1* KO BMDMs, MNV replication was not inhibited by IFNG, while ATG16L1 FL expression restored IFNG-mediated control of MNV replication (Fig. 3E and S1C). We observed that the ATG5 binding region and WIPI2 binding region (207-230) of ATG16L1 were essential for IFNG-mediated control of MNV in *Atg16l1* KO BMDMs (Fig. 3C and E). We next evaluated the ability of the ATG16L1mutants to promote the localization of LC3 with the MNV RC in infected cells. As expected, these results mirrored the IFNG inhibition of MNV replication in that the ΔATG5 binding region (i.e., the coiled-coil domain) and ΔWIPI2 binding (1-207) mutant of ATG16L1 did not restore the LC3 lipidation on MNV RC (Fig. 3F and Fig. S1D). Collectively, these data corroborate the importance of ATG5 binding region of ATG16L1 in LC3 lipidation on virus RC and MNV control by IFNG (8) and further demonstrate that the 208–230 region of ATG16L1 is required for LC3 conjugation to the viral RC and IFNG-mediated control of MNV.

### WIPI2 contributes to LC3 localization with MNC RC.

**FIG 4:**
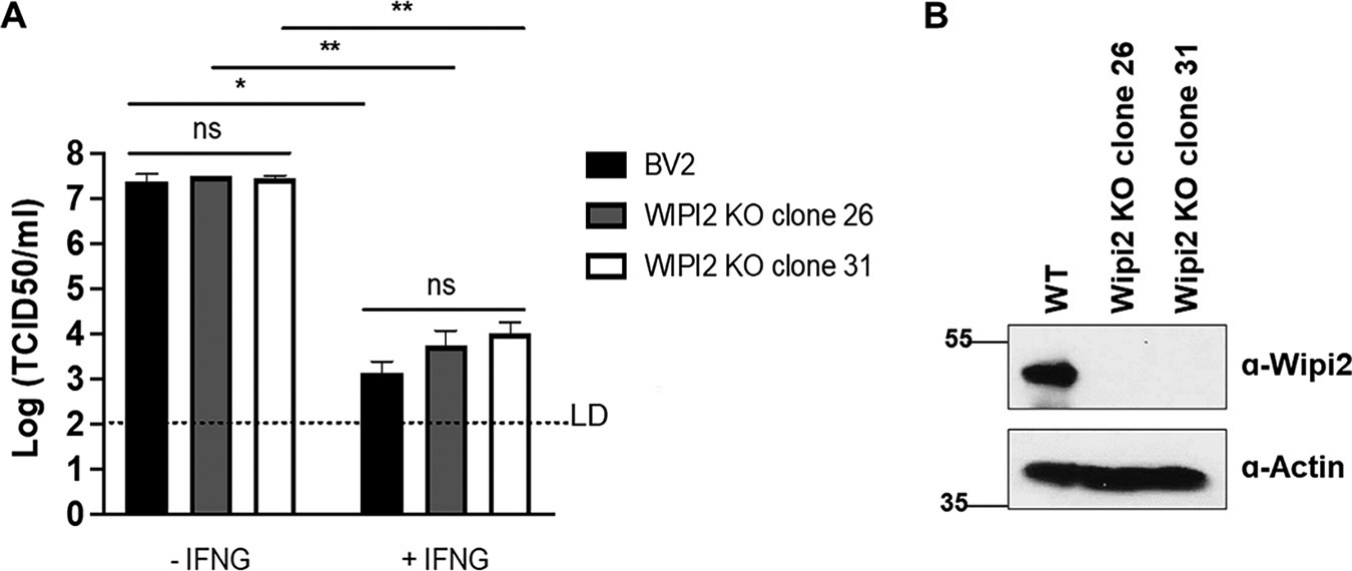
Wipi2 is not required for IFNG-mediated control of MNV replication. (A) MNV production in the WT and *Wipi2^−/−^* BV2 cells. The cells were untreated or treated with 100 U/mL of IFNG for 24 h and then infected with 0.05 MOI of MNV. At 24 hpi, cells were harvested to titer infectious virus by TCID_50_. (B) A representative Western blot data of cells described in Fig. 4A. Data were analyzed using *unpaired t test*. Ns, not significant; *, *P* < 0.05; **, *P* < 0.01. Dashed line indicates the limit of detection (LD).

The 208-230 region of ATG16L1 contains a WIPI2 interaction domain, which leads to recruitment of ATG16L1 to the membrane surrounding bacteria and subsequent restriction of bacterial proliferation (25). To test whether WIPI2 localizes with LC3 and the viral RC, we transfected MNV ORF1 and WIPI2-RFP into HeLa cells and examined the WIPI2-LC3-MNV Propol colocalization. Under the cotransfected condition, both WIPI2-RFP and LC3 localized on MNV RCs with high Mander’s coefficients (Fig. S2A). To test whether WIPI2 is required for LC3 colocalization with MNV RCs, WT and *Wipi2*^−/−^ mouse embryonic fibroblasts (MEFs) expressing an MNV receptor CD300LF (29, 30) were infected with MNV at MOI 10 for 10 h and fixed/stained with anti-Propol and anti-LC3 antibodies. Under these conditions, LC3 localization with MNV RC was observed in WT MEF; this localization was dependent on the presence of *Wipi2*, as their localizations were substantially reduced upon *Wipi2* deletion (Fig. S2B to D). Notably, some LC3 localization with MNV Propol remained in *Wipi2*^−/−^ MEFs, suggesting that WIPI2 was not absolutely required for LC3 colocalization with RC, and implicating a potential role of other ATG16L1-interacting proteins in RC targeting. Further, we examined the role of WIPI2 in IFNG-mediated inhibition of MNV replication. Here, we used BV2 cells, which can be readily infected by MNV through endogenous CD300LF. Intriguingly, MNV replication was controlled by IFNG in *Wipi2*^−/−^ BV2 cells, similar to the WT control (Fig. 4A and B). Thus, although *Wipi2* deletion resulted in decreased colocalization of LC3 with viral RC, it did not result in impaired IFNG control of MNV infection in BV2 cells, suggesting the possibility of functional redundancy.

10.1128/mbio.00172-23.2 FIG S2 Wipi2 colocalizes with the RC formed by MNV (A) IFA for the localization of Wipi2 and LC3 to viral RC. HeLa cells were transfected with MNV ORF1 and pDRFP-WIPI2 and then incubated for 24 hrs. The cells were fixed and stained with anti-Propol (RC) and anti-LC3 antibodies. Representative images shown here. Zoomed images shown for colocalization. Scale bar, 5 μm. Mander’s coefficients. (B) IFA for the lipidation of LC3 on MNV RC in WT and Wipi2^−/−^Cd300lf-MEF. Cells were infected with 10 MOI of MNV for 10 hrs and then fixed and stained with anti-Propol (RC) and anti-LC3 antibodies. Representative images shown here. Zoomed images shown for colocalization. Scale bar, 5 μm. (C) Quantification of IFA from (B). The result is shown as percentage of cells with LC3 on RC. (D) A representative western blot data showing expression of Wipi2 and Actin for the cells described in Fig. S2B and S2C. Download FIG S2, TIF file, 0.8 MB. Copyright © 2023 Kurhade et al. 2023 Kurhade et al. https://creativecommons.org/licenses/by/4.0/ This content is distributed under the terms of the Creative Commons Attribution 4.0 International license.

WIPI2 recruits the LC3 conjugation system to the autophagosome membranes through its association with phosphatidylinositol 3-phosphate (PI3P) enriched membranes (25). In addition, PI3P localized with the RCs of some RNA viruses, facilitating their replication (31, 32). We established *Cd300lf*-MEF cells stably expressing 2xFYVE-EGFP, which binds PI3P. PI3P production was significantly inhibited by wortmannin (a Pan-PI3K inhibitor that blocks PI3P production) compared with control cells (Fig. S3A), but we did not find any difference in LC3 localization with MNV RC upon wortmannin treatment (Fig. S3B and C). Further, we examined the role of IFNG in WT and VPS34-depleted MEFs, which is a major kinase for generating PI3P (33), using *Vps34^f/f^* MEFs (34). Consistent with the localization of LC3 independent of PI3P production, VPS34-deficiency did not show any effect on IFNG-mediated virus regulation (Fig. S3D and E). Collectively, these data show that WIPI2 is involved in LC3 localization onto the MNV RC but is not essential for IFNG-mediated MNV control.

10.1128/mbio.00172-23.3 FIG S3 PI3P is not essential for TAg. (A) IFA for the PI3P (2× FYVE-EGFP) in *Cd300lf-M*EF cells. *Cd300lf-M*EF cells were transduced with 2× FYVE-EGFP, a marker for the endogenous PI3P. Cells were then either left untreated or treated with wortmannin (200 nM) and fixed after 10 hrs. Representative images shown here. Scale bar, 20 μm. (B) LC3 lipidation on MNV RC in control and wortmannin treated 2× FYVE-EGFP *Cd300lf-M*EF cells. *Cd300lf-M*EF cells expressing 2× FYVE-EGFP were untreated or treated with wortmannin (200 nM) for 15 min and then inoculated with 5 MOI of MNV for 30 min. After 30 min inoculation, the medium was changed with wortmannin. After 10 hrs of infection/control, cells were fixed and stained with anti-Propol (RC) and anti-LC3 antibodies. (C) Quantification of IFA from (B). The result is shown as percentage of cells with LC3 on RC (± SEM). (D) Western blot showing the KD of Vps34 after transduction of EGFP (control) and Cre plasmid in *Vps34 flox/flox Cd300lf-M*EFs as described before (34) and actin control. (E) Growth analysis of MNV in untreated or IFNG treated WT and Vps34 KO *Cd300lf-M*EF cells. WT and Vps34 KO *Cd300lf-M*EF cells were either untreated or treated with 100 U/mL of IFNG for 24 h and then infected with 0.05 MOI of MNV. At 24 hpi, cells were harvested to titer infectious virus by TCID_50_ (*n* = 2). Download FIG S3, TIF file, 1.1 MB. Copyright © 2023 Kurhade et al. 2023 Kurhade et al. https://creativecommons.org/licenses/by/4.0/ This content is distributed under the terms of the Creative Commons Attribution 4.0 International license.

### The ATG16L1-interacting protein CAPRIN1 is important for IFNG-mediated control of MNV replication.

**FIG 5:**
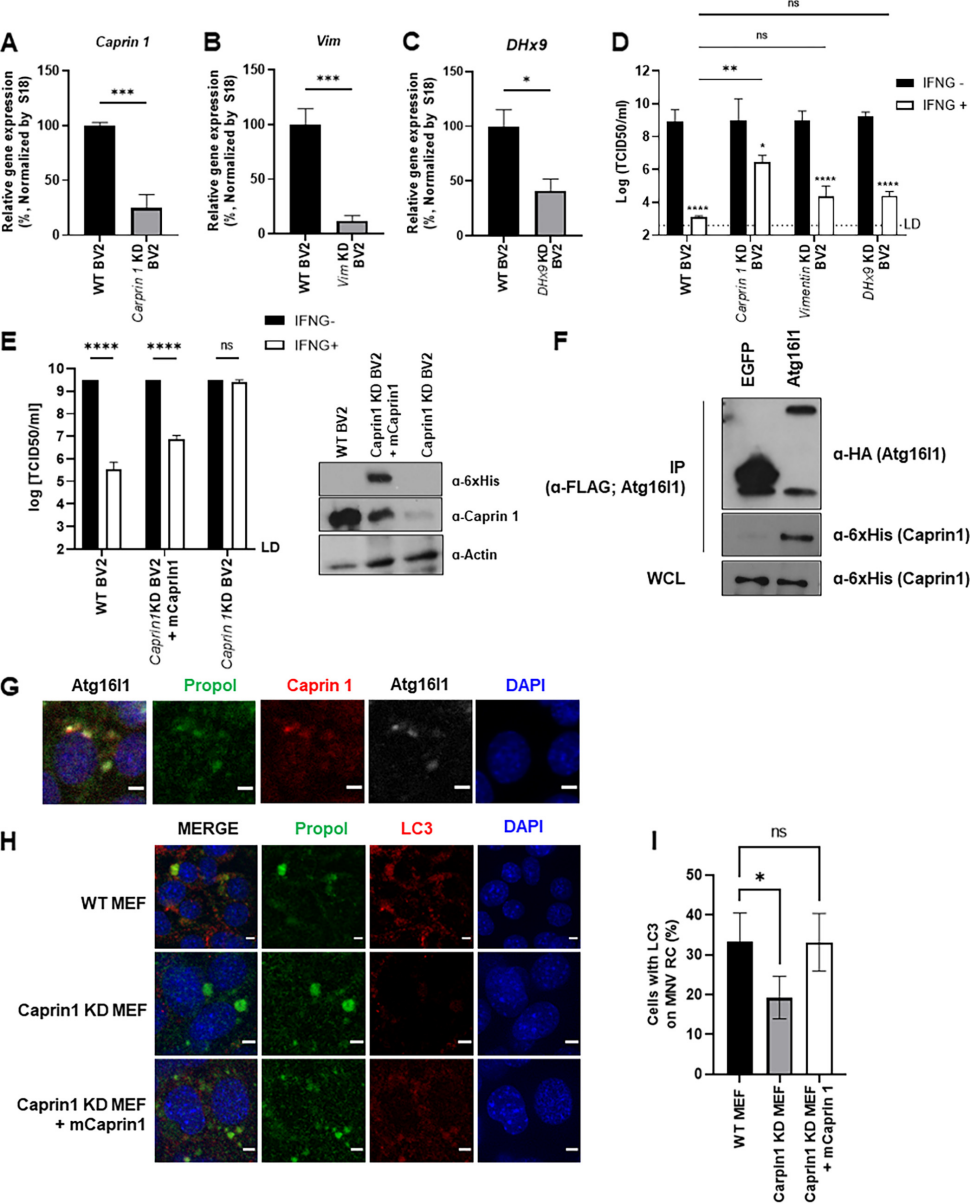
CAPRIN1 is required for LC3 targeting of RC and IFNG inhibition of MNV replication. (A-C) qRT-PCR of selected genes in control and shRNA-mediated gene knockdown (KD) in BV2 cells. BV2 cells were transduced with lentivirus expressing shRNA against *Caprin 1* (A), *Vim* (B), or *DHx9* (C) and then selected with puromycin (3 μg/mL) for 4 days. After selection, mRNA expression level from each KD cell lines were measured by qRT-PCR. Results are normalized to an average of control WT BV2 cells. (D) MNV production in untreated or IFNG treated WT and selected gene KD BV2 cells. WT and KD BV2 cells were untreated or treated with 100 U/mL of IFNG for 24 h and then infected with 0.05 MOI of MNV. At 24 hpi, cells and supernatants were harvested to titer infectious virus by TCID_50_. (E) MNV production in CAPRIN1 reconstituted BV2 cells. WT, *CAPRIN1* KD, and *CAPRIN1* KD expressing CAPRIN1 construct BV2 cells were untreated or treated with 100 U/mL of IFNG for 24 h and then infected with 0.05 MOI of MNV. At 24 hpi, cells and supernatants were harvested to titer infectious virus by TCID_50_. Representative Western blot of *CAPRIN1* KD and reconstituted BV2 cells is shown (E, right). (F) Representative Western blot analysis of a coimmunoprecipitation assay against FLAG-HA-ATG16L1 (FLAG) performed in extracts of 293T cells transfected with FLAG-HA-ATG16L1 and 6xHis-CAPRIN1. Levels of proteins are shown in whole-cell lysates (WCL) and FLAG-bound fractions. (G) IFA for localization of CAPRIN1 and ATG16L1 to viral RC. *Cd300lf-M*EF cells transduced with FLAG-HA-ATG16L1 and 6xHis-Caprin 1 were infected with 10 MOI of MNV. At 10 hpi, the cells were fixed and stained with antibodies for propol (RC), CAPRIN1 (6×-his), and ATG16L1 (HA). Representative images are shown. Scale bar, 5 μm. (H) IFA for the LC3 lipidation on viral RC in WT, *Caprin 1* KD, and CAPRIN1-reconstituted *Caprin 1* KD *Cd300lf-M*EF cell. *Cd300lf-M*EF cells were infected with 10 MOI of MNV. At 10 hpi, the cells were fixed and stained with anti-Propol (RC) and anti-LC3 antibodies. Representative images shown. Scale bar, 5 μm. (I) Quantification of IFA from (H). The result is shown as percentage of cells with LC3 on RC.

Given that WIPI2 was not essential for IFNG inhibition of MNV replication, we tested a role for other ATG16L1-interacting proteins in MNV-infected cells. To minimize identifying TAG-unrelated interacting proteins, we used the 1-230 ATG16L1 truncation mutant, which was functional for LC3 conjugation on RC and IFNG-mediated MNV control (Fig. 3E). We performed immunoprecipitation-mass spectrometry (IP-MS) (35) and identified proteins interacting with 1–230 ATG16L1 in uninfected and MNV-infected BV2 cells. We listed proteins found in both samples and highlighted the unique proteins that showed significantly higher signal from the MNV infected sample (Table S1). Among these proteins, we tested CAPRIN1, Vimentin and DHX9 for relevance in IFNG-mediated MNV control since they have been previously implicated in RC formation or function (36–48). Knockdown (KD) of Vimentin and DHX9 had no significant effect on IFNG inhibition of MNV replication; however, KD of CAPRIN1 significantly reduced the IFNG inhibition of MNV by ~1,000-fold (Fig. 5A to D). The failure of MNV control by IFNG in shCAPRIN1 cells was restored by reconstitution CAPRIN1 expression, thus ruling out off-target effects (Fig. 5E).

10.1128/mbio.00172-23.4 TABLE S1 ATG16L1 1-230 interacting proteins from IP-MS. Download Table S1, XLSX file, 0.08 MB. Copyright © 2023 Kurhade et al. 2023 Kurhade et al. https://creativecommons.org/licenses/by/4.0/ This content is distributed under the terms of the Creative Commons Attribution 4.0 International license.

It was recently reported that CAPRIN1 interacts with MNV RC during infection in replication-independent manner via its interacting partner, G3BP1 (49). Based on our IP-MS data with ATG16L1, we hypothesized that ATG16L1 complex might be recruited to the virus RC via interaction with CAPRIN1. We confirmed that ATG16L1 interacts with CAPRIN1 via co-IP (Fig. 5F) and further observed that CAPRIN1 colocalized with MNV RC and ATG16L1 (Fig. 5G). These data suggested that CAPRIN1 can bind to ATG16L1 and colocalize with MNV RC upon infection. Further, we found that LC3 localization with MNV RCs was dependent on CAPRIN1 expression and reduced LC3 localization on MNV RC by CAPRIN1 KD was successfully restored by CAPRIN1 reconstitution (Fig. 5H and I). Collectively, these results suggest that CAPRIN1 is required for LC3 localization with the MNV RC and IFNG-mediated control of MNV replication.

## DISCUSSION

In this study, we found that human hGBP1 is sufficient to localize with MNV RCs and to inhibit MNV replication upon IFNG stimulation. This is supportive of our previous finding that murine mGBP2, the homolog hGBP1, is required for IFNG control of MNV infection in the mouse system. The inability of hGBP1/mGBP2 to localize with MNV RCs in the absence of IFNG stimulation suggests that additional IFNG-induced factors are involved in recruiting GBPs to MNV RCs. The ability of GBP1 to inhibit MNV replication depends on its core properties of GTP binding, hydrolysis, and membrane binding. This suggests that dynamin-like activity of GBP, which multimerizes on the endomembranes and a pinches off small vesicles (50), plays a role in the control of RC via TAG. It should be noted that the relative contribution of the TAG system in IFNG-mediated control of MNV was more pronounced in murine cells than in human cells (e.g., Fig. 3E and 1D, respectively). This may reflect that human cells have an evolutionarily contracted IRG system (8, 51). How exactly GBP1 disrupts the MNV RC is the question of future investigation.

In autophagy, ATG16L1 determines the site of LC3 lipidation on the developing phagophore. The ATG16L1 protein, which has three main domains (N-terminus, coiled coil, and WD40 repeat), can drive LC3 lipidation on double-membrane autophagosomes and single membrane vesicles. We found that the ATG5-binding region of ATG16L1 was essential for LC3 lipidation of viral RC, which was expected, as the ATG12-ATG5-ATG16L1 complex as a whole is responsible for bringing LC3 to the developing phagophore (52). ATG16L1 is recruited to the developing phagophore by interacting with newly synthesized PI3P on the phagophore. This recruitment is mediated by either direct interaction of ATG16L1 with phosphoinositide or by binding to the PI3P effectors WIPI2 or FIP200 (24–27). We found that ATG16L1 region 207-230, which is known to bind WIPI2, is critical for recruiting ATG16L1 to the MNV RC, potentially implicating WIPI2 in RC recognition. We observed that WIPI2 localized to the MNV RC and affected LC3 localization with RCs, but intriguingly it was not required for IFNG-mediated control of MNV in BV2 cells. It is possible that the reduced level of LC3 localization with RCs in *WIPI2^−/−^* cells is still sufficient for IFNG control of MNV replication. Alternatively, WIPI2 function may be secondary to CAPRIN1 in IFNG control of MNV replication. Nevertheless, another study (53) suggested a crucial role of WIPI2 in the control of MNV replication by IFNG. This discrepancy might stem from differences in experimental systems. Unlike canonical autophagy, the PI3K activity, which promotes PI3P production, is not required for TAG-mediated IFNG control (9, 10); consistently, we found that VPS34 activity and PI3P were not required for TAG-dependent MNV control, demonstrating that this regulation is independent of PI3P. Overall, our data suggest that WIPI2 plays a crucial role in LC3 localization on MNV RC but is not essential for MNV inhibition by IFNG.

Our previous studies showed that LC3s are necessary to recruit the IFN-inducible GTPases to the RCs (8). Therefore, we speculated there might be a host protein that recruits ATG16L1 to the membrane of RCs and is required for IFNG inhibition of MNV replication. The conditions we considered to find a promising protein candidate were 1) a protein that interacts with ATG16L1 and 2) a protein that has the potential to interact with the replication compartment membrane at the same time. To explore the candidate proteins that meet these criteria, we searched the interactome of the functional ATG16L1 1-230 in MNV-infected cells. We found that CAPRIN1 is an ATG16L1-interacting protein required for TAG-dependent IFNG control of MNV infection. CAPRIN1, also known as RNA granule protein 105 (54), is an RNA-binding protein (RBP) and participates in stress granule (SG) formation through phosphorylation of eIF2α (55). CAPRIN1 regulates protein translation, cell migration, and proliferation in various cell types through interactions with several SG-associated RBPs, such as G3BPs and/or USP10 (55–57). Viruses interfere with host defense mechanisms against viral replication through binding with various RBPs, including CAPRIN1 (37, 58–61). During MNV infection, it was confirmed via IP that ATG16L1 interacts with CAPRIN1, which is presumed to be mediated by G3BP1, an interacting partner of CAPRIN1 (49, 62). Of note, CAPRIN1 interacts with ATG16L1 and G3BP1 in uninfected cells, and they are relocalized to MNV RC upon infection (49). We found that CAPRIN1 not only interacts with ATG16L1 but also is required for the IFN-mediated control of MNV replication.

The ATG16L1 1–230 interactome included several other RBPs in addition to CAPRIN-1. RNA viruses coopt and directly interact with host RBPs to affect the recruitment of viral genomes for replication, to help the membrane assembly for the replication compartment, to control RNA synthesis, and to make viral RNA stable (63). Since viral RC tagging by ATG16L1 is not limited to MNV, whether these RBPs, including CAPRIN-1, generally affect the TAG system is unclear. Also, CAPRIN1 involvement in the control of other (+) RNA virus RC, like encephalomyocarditis virus (8), needs further investigation.

**FIG 6:**
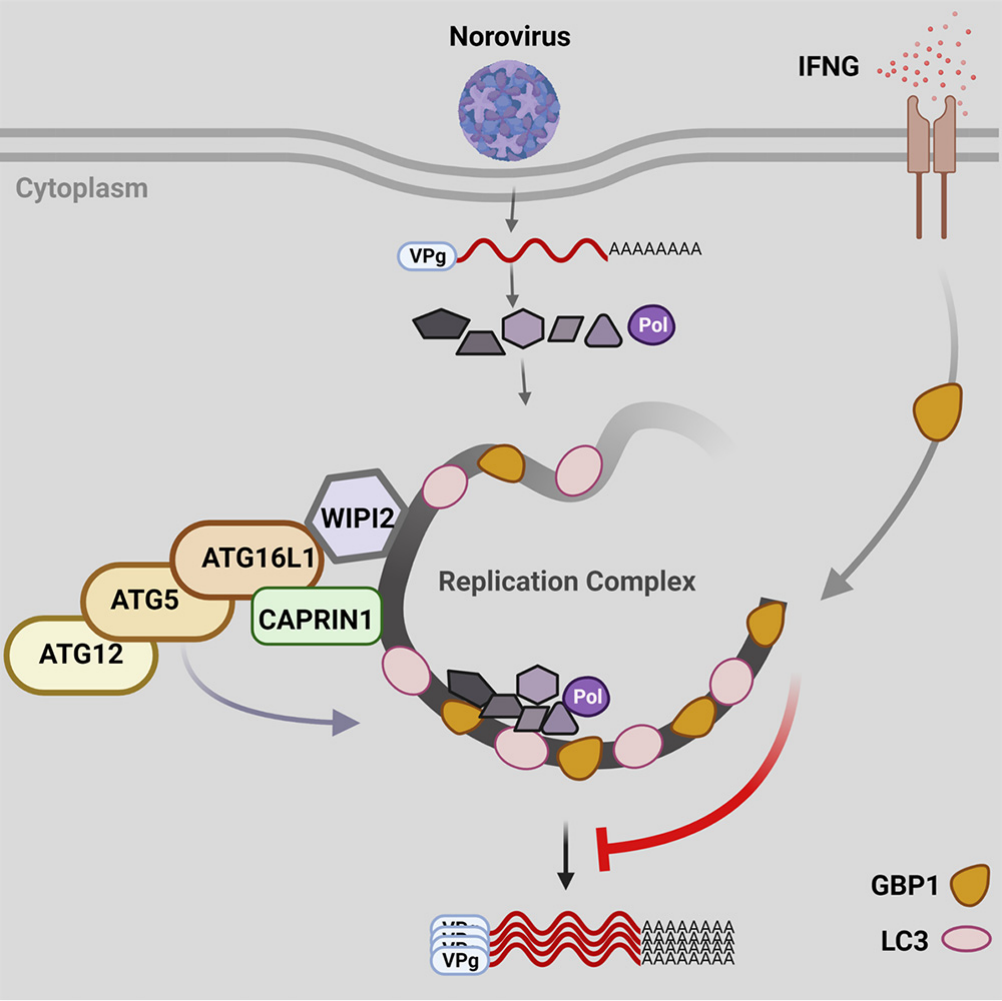
Model of RC recognition and inhibition by TAg. Viral RCs are affiliated with WIPI2 and CAPRIN1, which are bound by ATG16L1/AGT5/ATG12 to conjugate LC3 to RCs. INFG treatment induces the relocalization of hGBP1/mGBP2 to LC3-containing RCs, which promotes their destruction.

In summary, our study demonstrates that the ATG16L1 is a critical molecule to target the RC of the MNV and IFNG-mediated antiviral effect as a member of the LC3-conjugation system. This system was conserved in humans, targeting both MNV RC and HNV RC, and hGBP1 was sufficient to inhibit MNV replication by IFNG. Importantly, we identified CAPRIN1 as a previously unappreciated ATG16L1-interacting protein involved in recruiting the LC3 lipidation complex to RCs and required for the inhibition of MNV replication by IFNG (Fig. 6). This study warrants further investigations to understand the cell autonomous immune defense mechanism against viral RCs, the hallmark of all known (+) RNA viruses.

## MATERIALS AND METHODS

### Mice.

*Atg16l1^f/f^+LysMcre* mice is previously described (8). In all experiments, control and experimental mice were 6 to 8 week old littermates of both genders. Littermates of the same sex were housed together regardless of genotype. All mice were housed and bred at the University of Chicago under specific-pathogen-free conditions in a biosafety level 2 facility in accordance with federal and university guidelines. All experimental procedures were approved by the Animal Care and Use Committee of the University of Chicago.

### Primary cells.

The primary cells utilized in this study were murine bone marrow derived macrophages (BMDMs). Bone marrows were isolated from the femurs and tibias of 6 to 8 week old mice of both genders listed above and plated in nontissue culture treated 10-cm dishes in 10 mL of BMDM media. On day 4, 10 mL of fresh BMDM medium was added. On day 7, BMDMs were detached from the dish using ice-cold 0.02%EDTA in DPBS (Sigma-Aldrich, E8008), and seeded in tissue culture treated plates or on coverslips for subsequent experiments. Remaining D7 BMDMs were frozen in BMDM media containing 10%DMSO and used later to set up replicate experiments if necessary. BMDMs were rested for 3 days after seeding and used for experiments. The composition of BMDM medium was Dulbeco’s Modified Eagle Medium (Mediatech, 10-013), 10% fetal bovine serum (Biowest, US1520), 5% horse serum (Life Technology, 16050), 1× MEM nonessential amino acids (Mediatech, 25-025-CI), 1 mM sodium pyruvate (Mediatech, 25-000-CI), 2 mM l-glutamine (Mediatech, 25-005-CI), and macrophage colony-stimulating factor (M-CSF). The source of the M-CSF was 15% CMG14-12 conditioned medium (64) for MNV infection. All BMDMs were cultured at 37°C in 5% CO2. For *Atg16l1^f/f^ +LysMcre* mice BMDM were cultured for 25 days to completely KO the ATG16L1 in BMDM.

### Cell lines and transfection.

All continuous cell lines used in this study were grown in DMEM (Mediatech, 10-013) supplemented with 10 mM HEPES (Mediatech, 25-060-CI), 1× MEM nonessential amino acids (Mediatech, 25-025-CI), 100 U/mL each of penicillin and streptomycin (Mediatech, 30-002-CI) and 10% fetal bovine serum (Biowest, US1520) at 370C under 5% CO2. All transfections were done using Lipofectamine 3000 (Thermo Fischer-L3000001) as per the manufacturer’s instructions.

### CRISPR KOs of Wipi2 and GBP1.

The gene-specific guide RNA was cloned into lentiCRISPRv2 vector and cotransduced into BV2 cells with the packaging plasmids pVSVg and psPAX2. Forty-eight hours after transfection the lentiviral particles in the cell culture media were applied to cells for 48 h. The transduced cells were then selected with puromycin at 3 μg/mL for 48 to 72 h. Cells were seeded in single cell density and successful KO clones were confirmed by immunoblotting using antibody specific for Wipi2. For GBP1 KO in HeLa cells, gRNA was cloned in px458 (number 48138) and px459 (number 48139) plasmids that were obtained from Addgene. Cells were transfected with the plasmids using Lipofectamine 3000. After 48 h, transfected cells were enriched through one passage of puromycin (Sigma-Aldrich, P9620) treatment (3 μg/mL). Enriched cells were diluted and seeded in 96-well plates, aiming for 0.5 cell/well, and the monoclonal colonies of cells were picked at 7 days after the set-up and further amplified. The guide RNAs are listed in Table S2.

10.1128/mbio.00172-23.5 TABLE S2 Oligonucleotides used in this study. Download Table S2, XLSX file, 0.01 MB. Copyright © 2023 Kurhade et al. 2023 Kurhade et al. https://creativecommons.org/licenses/by/4.0/ This content is distributed under the terms of the Creative Commons Attribution 4.0 International license.

### Viral infection and transfection.

All MNV infections were conducted using the MNV-1.CW3 strain (8). MNV-1.CW3 viruses were prepared from a cDNA clone containing the genome of MNV-1.CW3. 1 × 10^6^ 293T cells were seeded in 6-well plates and transfected with 4 μg of the MNV-1.CW3 plasmid for 48 h to produce virus. MNV was further amplified in BV-2 cells. Infected cells were incubated until the cells showed > 90% cytopathic effect, usually for 48 h. Infected cells were then frozen and thawed, and the cell lysates containing viral particles were centrifuged for 20 min at 3000 rpm to remove the cell debris. Supernatants were further centrifuged for 3 h at 26,250 rpm at 4°C to produce a concentrated virus stock. The viral stocks were frozen in small aliquots, and the titers of the stocks were determined by TCID_50_ method as described earlier (8). All viral infections were conducted with a viral stock aliquot having undergone only one cycle of freeze and thaw. MNV vRNA was isolated from the concentrated MNV stocks using TRI reagent (Sigma-Aldrich, T9424) according to the manufacturer’s instruction. As human cells do not express MNV receptor, infection studies were conducted by transfecting MNV vRNA. The amount of vRNA indicated in figure legends was transfected to cells using Lipofectamine 3000 (Thermo Fischer-L3000001) as per manufacturer instruction manual.

### Lentiviral transduction.

All lentiviruses were generated in 293T cells as previously described (8). The lentiviral vector plasmids (psPAX2; Addgene, 12260) and pseudotyping vector (pMD2.G; Addgene, 12259) along with gene construct were transfected in 293T cells using the calcium phosphate precipitation method. Produced lentivirus was filtered through a 0.45 -μm syringe filter (Millipore, MA) and added onto the cells.

### TCID_50_ assay.

TCID_50_ assay was performed to quantitate infectious MNV as described previously (9). Serial 10-fold dilution of viral lysates were added to BV2 cells seeded in 96-well format. 8-wells were infected for each dilution and further incubated for 5 days. TCID_50_ was calculated by determining the dilution factor needed to show cytopathic effect (CPE) in 4 out of 8 (50%) wells. The limit of detection was calculated as the amount of virus that cause CPE in 4 out of 8 wells at the lowest dilution.

### Western blot.

Cells were harvested in sample buffer (0.1 M Tris [pH 6.8], 4% SDS, 4 mM EDTA, 286 mM 2-mercaptoethanol, 3.2 M glycerol, 0.05% bromophenol blue), and proteins were analyzed as previously described (8). Commercial antibodies used in this study are as follows: MNV Propol (9) ATG16L1 and LC3B (Sigma-Aldrich); GBP1-5 and Actin-HRP (Santa Cruz Biotechnology); HA (the Frank W. Fitch Monoclonal Antibody Facility, The University of Chicago); Flag-M2 (Thermo Fischer); WIPI2 (Abcam); HRP Goat anti-mouse and HRP Donkey anti-rabbit (Bio Legend); and HRP Donkey anti-goat (Jackson Immuno Research).

### Immunofluorescence’s analysis.

For immunofluorescence analysis cells were grown on cover glass (Fisher Scientific, PA; 12-545-80) in 24-well plates and then untreated/treated/infected as described in figure legends. Cells were fixed in 4% formaldehyde (Ted Pella, 18505), cells were permeabilized with 0.05% Saponin (Acros, 41923) in PBS, and blocked and probed in PBS containing 0.05% Saponin and 5% normal donkey serum (Jackson Immuno Research, 017-000-121). Fixation, permeabilization and 2 rounds of blocking were conducted for 10 min each at room temperature and staining with primary and secondary antibodies was conducted for 1 h each at room temperature. Samples were washed 5 times with PBS/0.01% Saponin for 5 min after each antibody incubation. Nuclei were stained with Hoechst 33342 (Invitrogen, H1399). Coverslips were mounted on glass slides with ProLong Diamond Antifade Mountant (Invitrogen, P36961). The images were acquired using Olympus DSU confocal microscope with a 60× water objective. Digital images were taken with Slidebook 6.0 software and images from each channel were merged using ImageJ (National Institutes of Health). Analysis of images was performed with ImageJ and used a set of defined intensity thresholds on all images. Primary antibodies detecting the following proteins were used: MNV Propol (9); FLAG (Sigma-Aldrich); LC3B (MBL International); GBP1-5 (Santa Cruz Biotechnology); HA (The Frank W. Fitch Monoclonal Antibody Facility, The University of Chicago); Rab11A (Thermo Fischer). DyLight 488 Goat polyclonal anti-Mouse (Biolegend); Alexa Fluor 488 Donkey polyclonal anti-Guinea Pig (Jackson ImmunoResearch); Alexa Fluor 555 Goat polyclonal anti-Mouse (Biolegend); Alexa Fluor 555 Donkey polyclonal anti-Rabbit (Biolegend); Dylight 649 Donkey polyclonal anti-Rabbit (Biolegend); Alexa Fluor 647 Donkey polyclonal anti-Guinea Pig (Jackson ImmunoResearch).

### qRT-PCR.

Total RNA was harvested from cells using RNeasy 96 kit (Qiagen) for measuring KD efficiency upon shRNA transduction. Cellular RNAs were reverse transcribed, and PCR amplified using the SuperScript III Platinum One-Step qRT-PCR System with Platinum *Taq* (Invitrogen) and IDT Primer Assays (Integrated DNA Technologies). Cellular RNAs were normalized to 18S levels using StepOnePlus System (Applied Biosystems).

### Mass spectrometry.

BV2 cell line stably expressing HA tagged 1–230 ATG16L1 truncation mutant was generated by lentiviral transduction. These cells were mock treated or infected with 10 MOI of MNV for 10 h. Cell lysate was pulled down on HA beads and sent to the Taplin Biological Mass Spectrometry Facility at Harvard Medical School for further analysis of interacting partners by mass spectrometry.

### Quantification and statistical analysis.

The mean with SD were calculated and described for all TCID50 and qRT-PCR data using two or more biological replicates. The Standard Error of Mean (SEM) were calculated and described for all IFA quantification and Mander’s coefficients calculation. All data were analyzed with Prism software (GraphPad) using one-way or two-way analysis of variation (One-way or two-way ANOVA) with multiple comparisons (for multiple samples), *unpaired t test* (for two samples) as indicated in figure legends. No specific method was used to determine whether the data met assumptions of the statistical approach. All differences not specifically indicated as significant were not significant (n.s., *P* > 0.05). Significant value was indicated as *, *P* < 0.05; **, *P* < 0.01; ***, *P* < 0.001; ****, *P* < 0.0001. Details of statistical significance and n values can be found in the figures or corresponding figure legends.

## References

[1] Woolhouse MEJ, Brierley L. 2018. Epidemiological characteristics of human-infective RNA viruses. Sci Data 5:180017. doi:10.1038/sdata.2018.17. 29461515 PMC5819479

[2] den Boon JA, Ahlquist P. 2010. Organelle-like membrane compartmentalization of positive-strand RNA virus replication factories. Annu Rev Microbiol 64:241–256. doi:10.1146/annurev.micro.112408.134012. 20825348

[3] Romero-Brey I, Bartenschlager R. 2014. Membranous replication factories induced by plus-strand RNA viruses. Viruses 6:2826–2857. doi:10.3390/v6072826. 25054883 PMC4113795

[4] Shulla A, Randall G. 2016. (+) RNA virus replication compartments: a safe home for (most) viral replication. Curr Opin Microbiol 32:82–88. doi:10.1016/j.mib.2016.05.003. 27253151 PMC4983521

[5] Mizushima N, Komatsu M. 2011. Autophagy: renovation of cells and tissues. Cell 147:728–741. doi:10.1016/j.cell.2011.10.026. 22078875

[6] Thukral L, Sengupta D, Ramkumar A, Murthy D, Agrawal N, Gokhale RS. 2015. The molecular mechanism underlying recruitment and insertion of lipid-anchored LC3 protein into membranes. Biophys J 109:2067–2078. doi:10.1016/j.bpj.2015.09.022. 26588566 PMC4656858

[7] Noda NN, Inagaki F. 2015. Mechanisms of autophagy. Annu Rev Biophys 44:101–122. doi:10.1146/annurev-biophys-060414-034248. 25747593

[8] Biering SB, Choi J, Halstrom RA, Brown HM, Beatty WL, Lee S, McCune BT, Dominici E, Williams LE, Orchard RC, Wilen CB, Yamamoto M, Coers J, Taylor GA, Hwang S. 2017. Viral replication complexes are targeted by LC3-guided interferon-inducible GTPases. Cell Host Microbe 22:74–85. doi:10.1016/j.chom.2017.06.005. 28669671 PMC5591033

[9] Hwang S, Maloney NS, Bruinsma MW, Goel G, Duan E, Zhang L, Shrestha B, Diamond MS, Dani A, Sosnovtsev SV, Green KY, Lopez-Otin C, Xavier RJ, Thackray LB, Virgin HW. 2012. Nondegradative role of Atg5-Atg12/Atg16L1 autophagy protein complex in antiviral activity of interferon gamma. Cell Host Microbe 11:397–409. doi:10.1016/j.chom.2012.03.002. 22520467 PMC3348177

[10] Choi J, Park S, Biering SB, Selleck E, Liu CY, Zhang X, Fujita N, Saitoh T, Akira S, Yoshimori T, Sibley LD, Hwang S, Virgin HW. 2014. The parasitophorous vacuole membrane of Toxoplasma gondii is targeted for disruption by ubiquitin-like conjugation systems of autophagy. Immunity 40:924–935. doi:10.1016/j.immuni.2014.05.006. 24931121 PMC4107903

[11] Park S, Choi J, Biering SB, Dominici E, Williams LE, Hwang S. 2016. Targeting by AutophaGy proteins (TAG): targeting of IFNG-inducible GTPases to membranes by the LC3 conjugation system of autophagy. Autophagy 12:1153–1167. doi:10.1080/15548627.2016.1178447. 27172324 PMC4990996

[12] Wheelock EF. 1965. Interferon-like virus-inhibitor induced in human leukocytes by phytohemagglutinin. Science 149:310–311. doi:10.1126/science.149.3681.310. 17533668

[13] Hoffmann HH, Schneider WM, Rice CM. 2015. Interferons and viruses: an evolutionary arms race of molecular interactions. Trends Immunol 36:124–138. doi:10.1016/j.it.2015.01.004. 25704559 PMC4384471

[14] Billiau A, Matthys P. 2009. Interferon-gamma: a historical perspective. Cytokine Growth Factor Rev 20:97–113. doi:10.1016/j.cytogfr.2009.02.004. 19268625

[15] Kang S, Brown HM, Hwang S. 2018. Direct antiviral mechanisms of interferon-gamma. Immune Netw 18:e33. doi:10.4110/in.2018.18.e33. 30402328 PMC6215902

[16] Bekpen C, Hunn JP, Rohde C, Parvanova I, Guethlein L, Dunn DM, Glowalla E, Leptin M, Howard JC. 2005. The interferon-inducible p47 (IRG) GTPases in vertebrates: loss of the cell autonomous resistance mechanism in the human lineage. Genome Biol 6:R92. doi:10.1186/gb-2005-6-11-r92. 16277747 PMC1297648

[17] Kravets E, Degrandi D, Ma Q, Peulen TO, Klumpers V, Felekyan S, Kuhnemuth R, Weidtkamp-Peters S, Seidel CA, Pfeffer K. 2016. Guanylate binding proteins directly attack Toxoplasma gondii via supramolecular complexes. Elife 5:e11479. doi:10.7554/eLife.11479. 26814575 PMC4786432

[18] Piro AS, Hernandez D, Luoma S, Feeley EM, Finethy R, Yirga A, Frickel EM, Lesser CF, Coers J. 2017. Detection of cytosolic shigella flexneri via a C-terminal triple-arginine motif of GBP1 inhibits actin-based motility. mBio 8(6):e01979-17. doi:10.1128/mBio.01979-17. 29233899 PMC5727416

[19] Britzen-Laurent N, Bauer M, Berton V, Fischer N, Syguda A, Reipschlager S, Naschberger E, Herrmann C, Sturzl M. 2010. Intracellular trafficking of guanylate-binding proteins is regulated by heterodimerization in a hierarchical manner. PLoS One 5:e14246. doi:10.1371/journal.pone.0014246. 21151871 PMC2998424

[20] Tretina K, Park ES, Maminska A, MacMicking JD. 2019. Interferon-induced guanylate-binding proteins: guardians of host defense in health and disease. J Exp Med 216:482–500. doi:10.1084/jem.20182031. 30755454 PMC6400534

[21] Ohshima J, Lee Y, Sasai M, Saitoh T, Su Ma J, Kamiyama N, Matsuura Y, Pann-Ghill S, Hayashi M, Ebisu S, Takeda K, Akira S, Yamamoto M. 2014. Role of mouse and human autophagy proteins in IFN-γ-induced cell-autonomous responses against Toxoplasma gondii. J Immunol 192:3328–3335. doi:10.4049/jimmunol.1302822. 24563254

[22] Parkhouse R, Ebong IO, Robinson CV, Monie TP. 2013. The N-terminal region of the human autophagy protein ATG16L1 contains a domain that folds into a helical structure consistent with formation of a coiled-coil. PLoS One 8:e76237. doi:10.1371/journal.pone.0076237. 24086718 PMC3782427

[23] Doerflinger SY, Cortese M, Romero-Brey I, Menne Z, Tubiana T, Schenk C, White PA, Bartenschlager R, Bressanelli S, Hansman GS, Lohmann V. 2017. Membrane alterations induced by nonstructural proteins of human norovirus. PLoS Pathog 13:e1006705. doi:10.1371/journal.ppat.1006705. 29077760 PMC5678787

[24] Lystad AH, Carlsson SR, de la Ballina LR, Kauffman KJ, Nag S, Yoshimori T, Melia TJ, Simonsen A. 2019. Distinct functions of ATG16L1 isoforms in membrane binding and LC3B lipidation in autophagy-related processes. Nat Cell Biol 21:372–383. doi:10.1038/s41556-019-0274-9. 30778222 PMC7032593

[25] Dooley HC, Razi M, Polson HE, Girardin SE, Wilson MI, Tooze SA. 2014. WIPI2 links LC3 conjugation with PI3P, autophagosome formation, and pathogen clearance by recruiting Atg12-5-16L1. Mol Cell 55:238–252. doi:10.1016/j.molcel.2014.05.021. 24954904 PMC4104028

[26] Gammoh N. 2020. The multifaceted functions of ATG16L1 in autophagy and related processes. J Cell Sci 133 doi:10.1242/jcs.249227. 33127840

[27] Nishimura T, Kaizuka T, Cadwell K, Sahani MH, Saitoh T, Akira S, Virgin HW, Mizushima N. 2013. FIP200 regulates targeting of Atg16L1 to the isolation membrane. EMBO Rep 14:284–291. doi:10.1038/embor.2013.6. 23392225 PMC3589088

[28] Gammoh N, Florey O, Overholtzer M, Jiang X. 2013. Interaction between FIP200 and ATG16L1 distinguishes ULK1 complex-dependent and -independent autophagy. Nat Struct Mol Biol 20:144–149. doi:10.1038/nsmb.2475. 23262492 PMC3565010

[29] Graziano VR, Walker FC, Kennedy EA, Wei J, Ettayebi K, Strine MS, Filler RB, Hassan E, Hsieh LL, Kim AS, Kolawole AO, Wobus CE, Lindesmith LC, Baric RS, Estes MK, Orchard RC, Baldridge MT, Wilen CB. 2020. CD300lf is the primary physiologic receptor of murine norovirus but not human norovirus. PLoS Pathog 16:e1008242. doi:10.1371/journal.ppat.1008242. 32251490 PMC7162533

[30] Haga K, Fujimoto A, Takai-Todaka R, Miki M, Doan YH, Murakami K, Yokoyama M, Murata K, Nakanishi A, Katayama K. 2016. Functional receptor molecules CD300lf and CD300ld within the CD300 family enable murine noroviruses to infect cells. Proc Natl Acad Sci USA 113:E6248–E6255. doi:10.1073/pnas.1605575113. 27681626 PMC5068309

[31] Gimenez MC, Issa M, Sheth J, Colombo MI, Terebiznik MR, Delgui LR. 2021. Phosphatidylinositol 3-phosphate mediates the establishment of infectious bursal disease virus replication complexes in association with early endosomes. J Virol 95. doi:10.1128/JVI.02313-20. PMC8094959 33361427

[32] Feng Z, Xu K, Kovalev N, Nagy PD. 2019. Recruitment of Vps34 PI3K and enrichment of PI3P phosphoinositide in the viral replication compartment is crucial for replication of a positive-strand RNA virus. PLoS Pathog 15:e1007530. doi:10.1371/journal.ppat.1007530. 30625229 PMC6342326

[33] Balla T. 2013. Phosphoinositides: tiny lipids with giant impact on cell regulation. Physiol Rev 93:1019–1137. doi:10.1152/physrev.00028.2012. 23899561 PMC3962547

[34] Jaber N, Mohd-Naim N, Wang Z, DeLeon JL, Kim S, Zhong H, Sheshadri N, Dou Z, Edinger AL, Du G, Braga VM, Zong WX. 2016. Vps34 regulates Rab7 and late endocytic trafficking through recruitment of the GTPase-activating protein Armus. J Cell Sci 129:4424–4435. 27793976 10.1242/jcs.192260 PMC5201010

[35] Valdez-Sinon AN, Gokhale A, Faundez V, Bassell GJ. 2020. Protocol for immuno-enrichment of FLAG-tagged protein complexes. STAR Protoc 1:100083. doi:10.1016/j.xpro.2020.100083. 33111116 PMC7580096

[36] Hou S, Kumar A, Xu Z, Airo AM, Stryapunina I, Wong CP, Branton W, Tchesnokov E, Götte M, Power C, Hobman TC. 2017. Zika virus hijacks stress granule proteins and modulates the host stress response. J Virol 91 doi:10.1128/JVI.00474-17. PMC5533921 28592527

[37] Katoh H, Okamoto T, Fukuhara T, Kambara H, Morita E, Mori Y, Kamitani W, Matsuura Y. 2013. Japanese encephalitis virus core protein inhibits stress granule formation through an interaction with Caprin-1 and facilitates viral propagation. J Virol 87:489–502. doi:10.1128/JVI.02186-12. 23097442 PMC3536427

[38] Bidet K, Dadlani D, Garcia-Blanco MA. 2014. G3BP1, G3BP2 and CAPRIN1 are required for translation of interferon stimulated mRNAs and are targeted by a dengue virus non-coding RNA. PLoS Pathog 10:e1004242. doi:10.1371/journal.ppat.1004242. 24992036 PMC4081823

[39] Katsafanas GC, Moss B. 2007. Colocalization of transcription and translation within cytoplasmic poxvirus factories coordinates viral expression and subjugates host functions. Cell Host Microbe 2:221–228. doi:10.1016/j.chom.2007.08.005. 18005740 PMC2084088

[40] Bonenfant G, Williams N, Netzband R, Schwarz MC, Evans MJ, Pager CT. 2019. Zika virus subverts stress granules to promote and restrict viral gene expression. J Virol 93(12):e00520-19. doi:10.1128/JVI.00520-19. 30944179 PMC6613768

[41] Ward AM, Gunaratne J, Garcia-Blanco MA. 2014. Identification of dengue RNA binding proteins using RNA chromatography and quantitative mass spectrometry. Methods Mol Biol 1138:253–270. doi:10.1007/978-1-4939-0348-1_16. 24696342

[42] Katsafanas GC, Moss B. 2004. Vaccinia virus intermediate stage transcription is complemented by Ras-GTPase-activating protein SH3 domain-binding protein (G3BP) and cytoplasmic activation/proliferation-associated protein (p137) individually or as a heterodimer. J Biol Chem 279:52210–52217. doi:10.1074/jbc.M411033200. 15471883

[43] Teo CS, Chu JJ. 2014. Cellular vimentin regulates construction of dengue virus replication complexes through interaction with NS4A protein. J Virol 88:1897–1913. doi:10.1128/JVI.01249-13. 24284321 PMC3911532

[44] Du N, Cong H, Tian H, Zhang H, Zhang W, Song L, Tien P. 2014. Cell surface vimentin is an attachment receptor for enterovirus 71. J Virol 88:5816–5833. doi:10.1128/JVI.03826-13. 24623428 PMC4019121

[45] Liang JJ, Yu CY, Liao CL, Lin YL. 2011. Vimentin binding is critical for infection by the virulent strain of Japanese encephalitis virus. Cell Microbiol 13:1358–1370. doi:10.1111/j.1462-5822.2011.01624.x. 21707907

[46] Yang J, Zou L, Yang Y, Yuan J, Hu Z, Liu H, Peng H, Shang W, Zhang X, Zhu J, Rao X. 2016. Superficial vimentin mediates DENV-2 infection of vascular endothelial cells. Sci Rep 6:38372. doi:10.1038/srep38372. 27910934 PMC5133558

[47] Yu YT, Chien SC, Chen IY, Lai CT, Tsay YG, Chang SC, Chang MF. 2016. Surface vimentin is critical for the cell entry of SARS-CoV. J Biomed Sci 23:14. doi:10.1186/s12929-016-0234-7. 26801988 PMC4724099

[48] Matkovic R, Bernard E, Fontanel S, Eldin P, Chazal N, Hassan Hersi D, Merits A, Péloponèse JM, Jr, Briant L. 2019. The host DHX9 DExH-box helicase is recruited to Chikungunya virus replication complexes for optimal genomic RNA translation. J Virol 93. doi:10.1128/JVI.01764-18. PMC6364007 30463980

[49] Brocard M, Iadevaia V, Klein P, Hall B, Lewis G, Lu J, Burke J, Willcocks MM, Parker R, Goodfellow IG, Ruggieri A, Locker N. 2020. Norovirus infection results in eIF2α independent host translation shut-off and remodels the G3BP1 interactome evading stress granule formation. PLoS Pathog 16:e1008250. doi:10.1371/journal.ppat.1008250. 31905230 PMC6964919

[50] Hinshaw JE. 2000. Dynamin and its role in membrane fission. Annu Rev Cell Dev Biol 16:483–519. doi:10.1146/annurev.cellbio.16.1.483. 11031245 PMC4781412

[51] Bekpen C, Marques-Bonet T, Alkan C, Antonacci F, Leogrande MB, Ventura M, Kidd JM, Siswara P, Howard JC, Eichler EE. 2009. Death and resurrection of the human IRGM gene. PLoS Genet 5:e1000403. doi:10.1371/journal.pgen.1000403. 19266026 PMC2644816

[52] Kharaziha P, Panaretakis T. 2017. Dynamics of Atg5-Atg12-Atg16L1 aggregation and deaggregation. Methods Enzymol 587:247–255. doi:10.1016/bs.mie.2016.09.059. 28253959

[53] McAllaster MR, Bhushan J, Balce DR, Orvedahl A, Park A, Hwang S, Sullender ME, Sibley LD, Virgin HW. 2021. Autophagy gene-dependent intracellular immunity triggered by interferon-γ. bioRxiv. doi:10.1101/2021.05.10.443539. PMC10746157 37905813

[54] Grill B, Wilson GM, Zhang KX, Wang B, Doyonnas R, Quadroni M, Schrader JW. 2004. Activation/division of lymphocytes results in increased levels of cytoplasmic activation/proliferation-associated protein-1: prototype of a new family of proteins. J Immunol 172:2389–2400. doi:10.4049/jimmunol.172.4.2389. 14764709

[55] Solomon S, Xu Y, Wang B, David MD, Schubert P, Kennedy D, Schrader JW. 2007. Distinct structural features of caprin-1 mediate its interaction with G3BP-1 and its induction of phosphorylation of eukaryotic translation initiation factor 2alpha, entry to cytoplasmic stress granules, and selective interaction with a subset of mRNAs. Mol Cell Biol 27:2324–2342. doi:10.1128/MCB.02300-06. 17210633 PMC1820512

[56] Soncini C, Berdo I, Draetta G. 2001. Ras-GAP SH3 domain binding protein (G3BP) is a modulator of USP10, a novel human ubiquitin specific protease. Oncogene 20:3869–3879. doi:10.1038/sj.onc.1204553. 11439350

[57] Shiina N, Shinkura K, Tokunaga M. 2005. A novel RNA-binding protein in neuronal RNA granules: regulatory machinery for local translation. J Neurosci 25:4420–4434. doi:10.1523/JNEUROSCI.0382-05.2005. 15858068 PMC6725113

[58] Ng CS, Jogi M, Yoo JS, Onomoto K, Koike S, Iwasaki T, Yoneyama M, Kato H, Fujita T. 2013. Encephalomyocarditis virus disrupts stress granules, the critical platform for triggering antiviral innate immune responses. J Virol 87:9511–9522. doi:10.1128/JVI.03248-12. 23785203 PMC3754122

[59] Emara MM, Brinton MA. 2007. Interaction of TIA-1/TIAR with West Nile and dengue virus products in infected cells interferes with stress granule formation and processing body assembly. Proc Natl Acad Sci USA 104:9041–9046. doi:10.1073/pnas.0703348104. 17502609 PMC1885624

[60] Scholte FE, Tas A, Albulescu IC, Žusinaite E, Merits A, Snijder EJ, van Hemert MJ. 2015. Stress granule components G3BP1 and G3BP2 play a proviral role early in Chikungunya virus replication. J Virol 89:4457–4469. doi:10.1128/JVI.03612-14. 25653451 PMC4442398

[61] Rabouw HH, Langereis MA, Knaap RC, Dalebout TJ, Canton J, Sola I, Enjuanes L, Bredenbeek PJ, Kikkert M, de Groot RJ, van Kuppeveld FJ. 2016. Middle East respiratory coronavirus accessory protein 4a inhibits PKR-mediated antiviral stress responses. PLoS Pathog 12:e1005982. doi:10.1371/journal.ppat.1005982. 27783669 PMC5081173

[62] Hosmillo M, Lu J, McAllaster MR, Eaglesham JB, Wang X, Emmott E, Domingues P, Chaudhry Y, Fitzmaurice TJ, Tung MK, Panas MD, McInerney G, Locker N, Wilen CB, Goodfellow IG. 2019. Noroviruses subvert the core stress granule component G3BP1 to promote viral VPg-dependent translation. Elife 8. doi:10.7554/eLife.46681. PMC6739877 31403400

[63] Li Z, Nagy PD. 2011. Diverse roles of host RNA binding proteins in RNA virus replication. RNA Biol 8:305–315. doi:10.4161/rna.8.2.15391. 21505273 PMC3230553

[64] Takeshita S, Kaji K, Kudo A. 2000. Identification and characterization of the new osteoclast progenitor with macrophage phenotypes being able to differentiate into mature osteoclasts. J Bone Miner Res 15:1477–1488. doi:10.1359/jbmr.2000.15.8.1477. 10934646

