# Supplementary figures and images for: CAPRIN1 Is Required for Control of Viral Replication Complexes by Interferon Gamma
Source: mBio. 2023 Apr 13;14(3):e00172-23. doi: 10.1128/mbio.00172-23 (PMC10294620; doi:10.1128/mbio.00172-23)

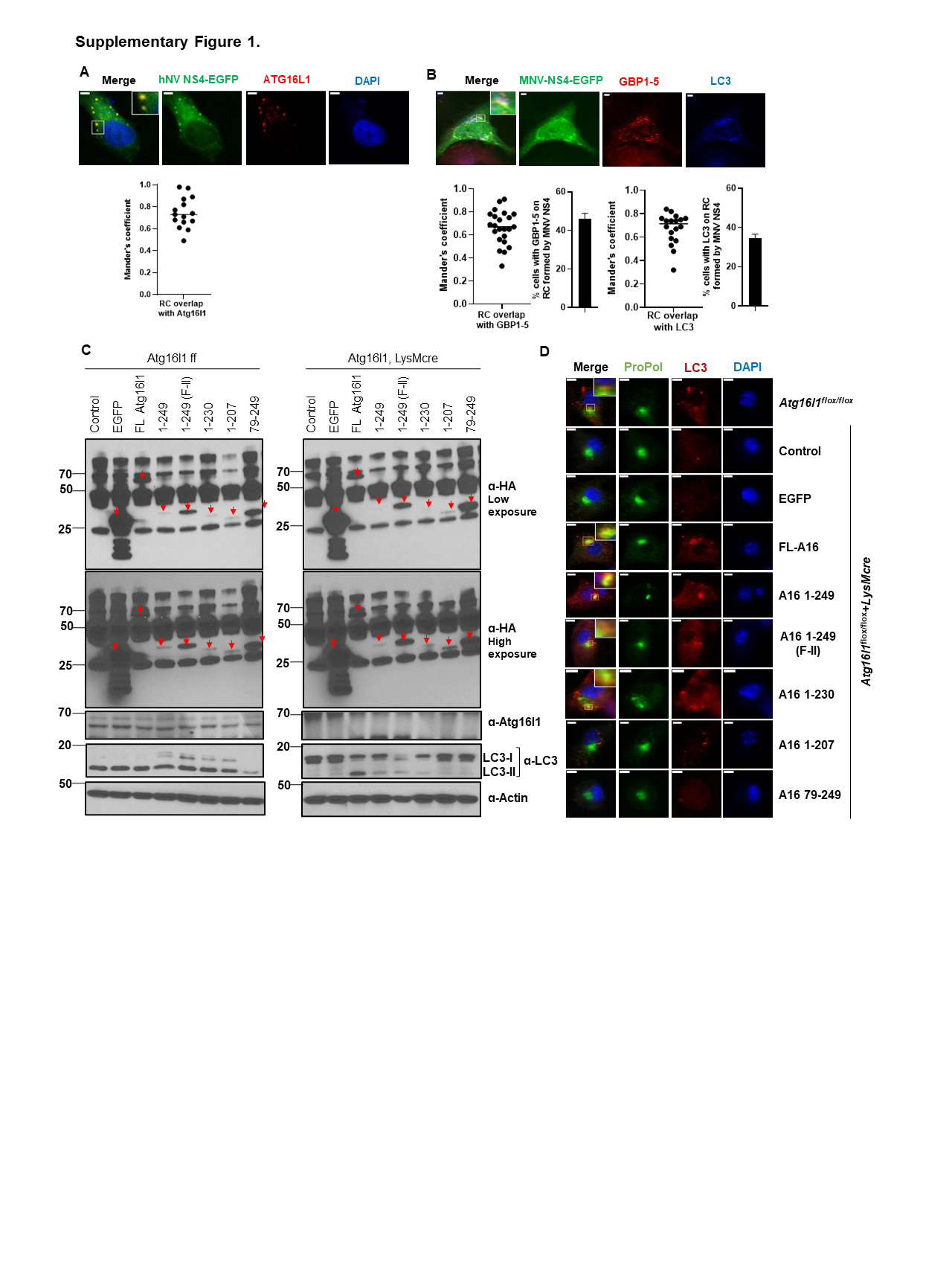

Supplement: FIG S1 [file mbio.00172-23-s0002.tif]

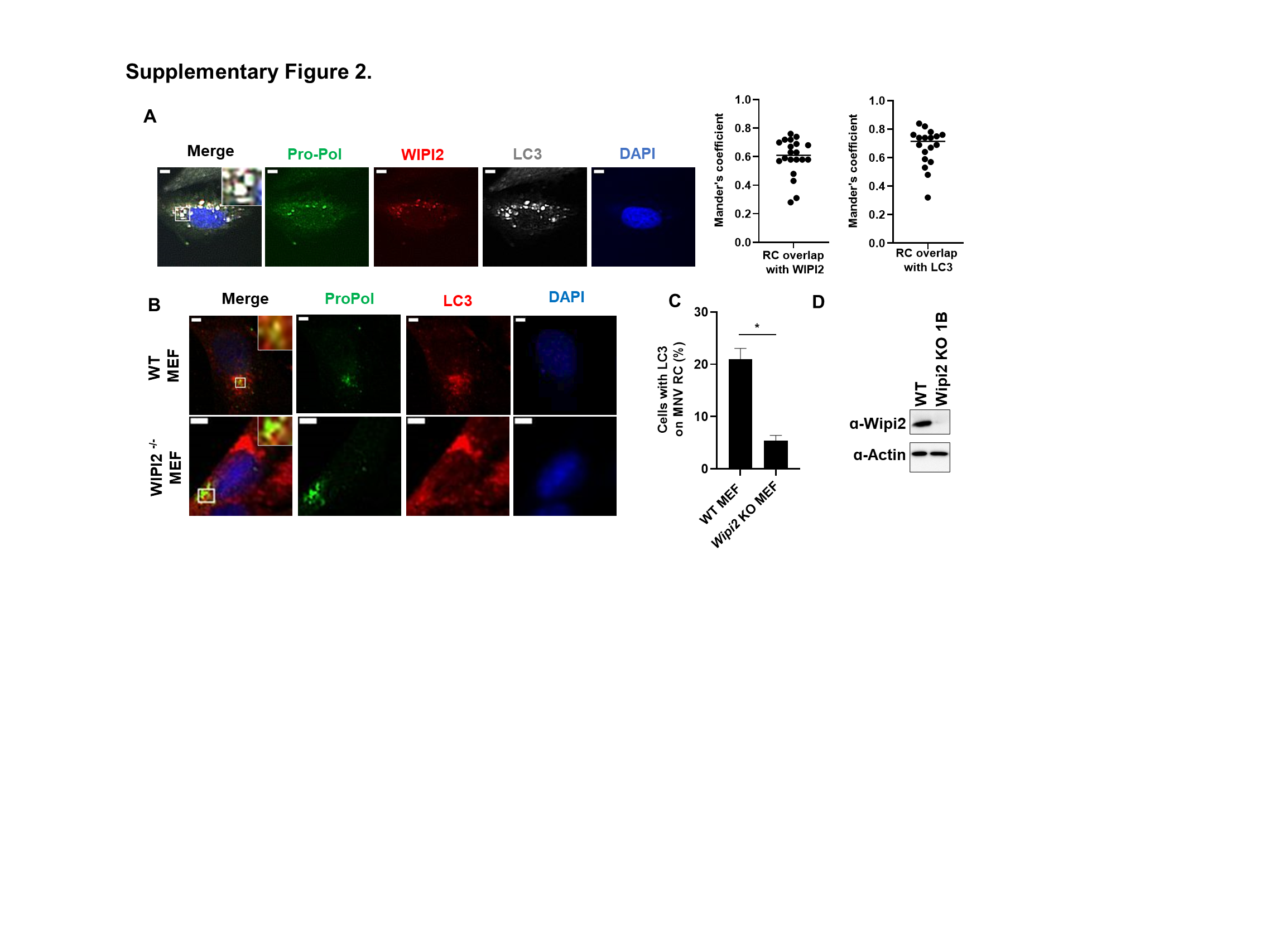

Supplement: FIG S2 [file mbio.00172-23-s0003.tif]

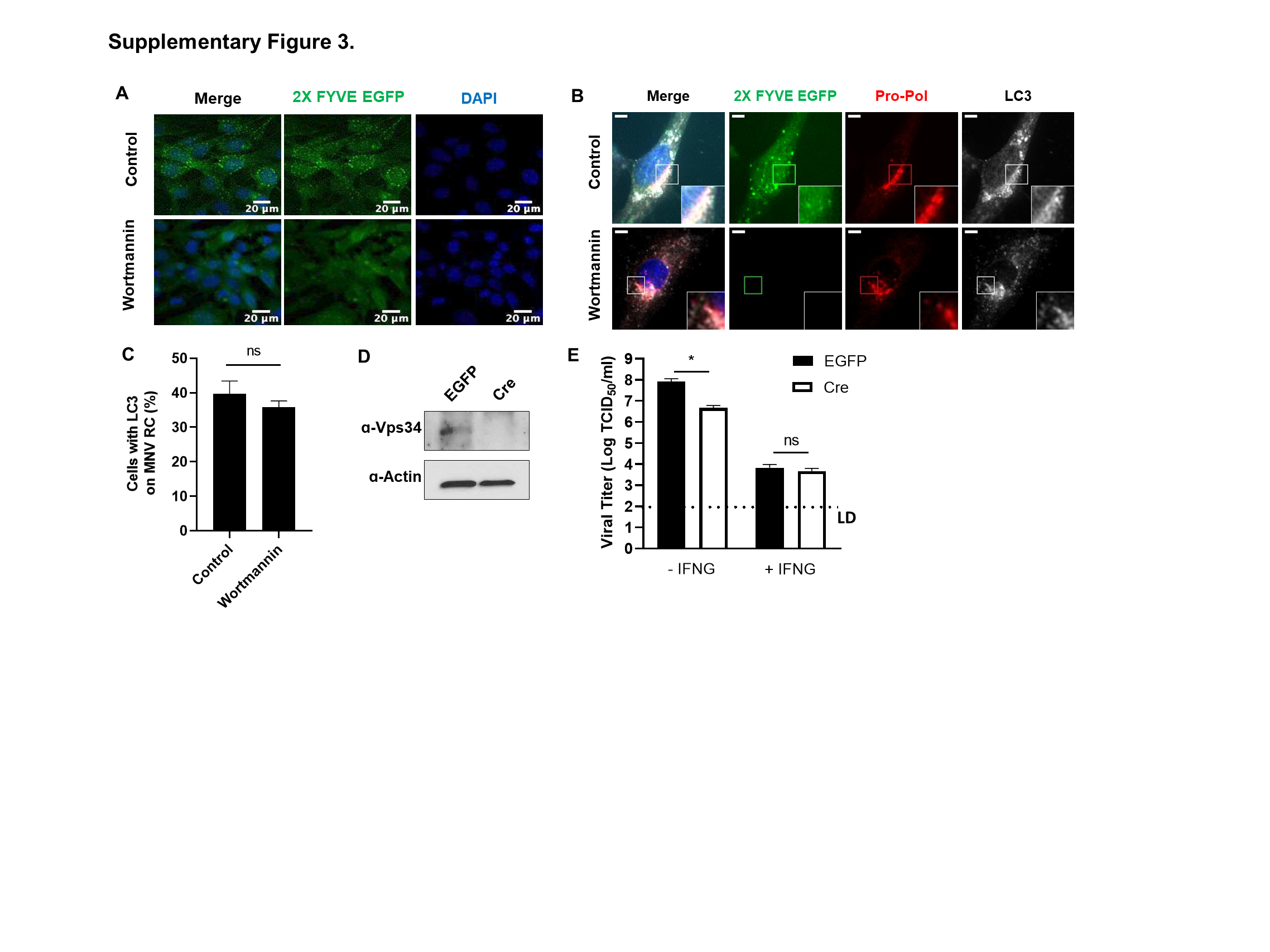

Supplement: FIG S3 [file mbio.00172-23-s0004.tif]
